# Supplementary material for: HIVprotI: an integrated web based platform for prediction and design of HIV proteins inhibitors
Source: J Cheminform. 2018 Mar 9;10:12. doi: 10.1186/s13321-018-0266-y (PMC5845081; doi:10.1186/s13321-018-0266-y)
Supplement: Supplementary file 2 — Additional file 2. Source code of HIVProtI web server. [file 13321_2018_266_MOESM2_ESM.zip › HIVprotI_Source-code/hivprotI/weka-3-6-6/doc/weka/classifiers/functions/supportVector/SMOset.html]

SMOset


---


|  |  |  |
| --- | --- | --- |
| **Overview** \| **Package** \| **Class** \| **Tree** \| **Deprecated** \| **Index** \| **Help** \| **Weka's home** | |  |
| **PREV CLASS**   **NEXT CLASS** | **FRAMES**    **NO FRAMES**     **All Classes** |  |
| SUMMARY: NESTED \| FIELD \| CONSTR \| METHOD | DETAIL: FIELD \| CONSTR \| METHOD |  |


---


## weka.classifiers.functions.supportVector Class SMOset

```
java.lang.Object
  weka.classifiers.functions.supportVector.SMOset
```

**All Implemented Interfaces:**: java.io.Serializable, RevisionHandler

---

``` public class SMOset extends java.lang.Object implements java.io.Serializable, RevisionHandler ```

Stores a set of integer of a given size.

**Version:**
:   $Revision: 1.5 $

**Author:**
:   Eibe Frank (eibe@cs.waikato.ac.nz)

**See Also:**: Serialized Form

---

**Constructor Summary**

`SMOset(int size)`
  
          Creates a new set of the given size.


| **Method Summary** | |
| --- | --- |
| `boolean` | `contains(int index)`             Checks whether an element is in the set. |
| `void` | `delete(int index)`             Deletes an element from the set. |
| `int` | `getNext(int index)`             Gets the next element in the set. |
| `java.lang.String` | `getRevision()`             Returns the revision string. |
| `void` | `insert(int index)`             Inserts an element into the set. |
| `int` | `numElements()`             Returns the number of elements in the set. |
| `void` | `printElements()`             Prints all the current elements in the set. |

**Methods inherited from class java.lang.Object**

`equals, getClass, hashCode, notify, notifyAll, toString, wait, wait, wait`

**Constructor Detail**

### SMOset

```
public SMOset(int size)
```

:   Creates a new set of the given size.


**Method Detail**

### contains

```
public boolean contains(int index)
```

:   Checks whether an element is in the set.

---


### delete

```
public void delete(int index)
```

:   Deletes an element from the set.

---


### insert

```
public void insert(int index)
```

:   Inserts an element into the set.

---


### getNext

```
public int getNext(int index)
```

:   Gets the next element in the set. -1 gets the first one.

---


### printElements

```
public void printElements()
```

:   Prints all the current elements in the set.

---


### numElements

```
public int numElements()
```

:   Returns the number of elements in the set.

---


### getRevision

```
public java.lang.String getRevision()
```

:   Returns the revision string.

    :   **Specified by:**: `getRevision` in interface `RevisionHandler`
    :   **Returns:**: the revision


---


|  |  |  |
| --- | --- | --- |
| **Overview** \| **Package** \| **Class** \| **Tree** \| **Deprecated** \| **Index** \| **Help** \| **Weka's home** | |  |
| **PREV CLASS**   **NEXT CLASS** | **FRAMES**    **NO FRAMES**     **All Classes** |  |
| SUMMARY: NESTED \| FIELD \| CONSTR \| METHOD | DETAIL: FIELD \| CONSTR \| METHOD |  |


---
